# Surviving but not thriving: Comparing primary, vocational and higher education teachers’ experiences during the COVID-19 lockdown

**DOI:** 10.1007/s10639-021-10616-x

**Published:** 2021-06-22

**Authors:** Helena Kovacs, Caroline Pulfrey, Emilie-Charlotte Monnier

**Affiliations:** 1 Center for Learning Sciences LEARN, EPFL, Lausanne, Switzerland; 2 EPFL, University of Lausanne, Haute École Pédagogique du Canton Vaud, Lausanne, Switzerland

**Keywords:** Comparative research, Teachers' experiences, COVID-19 lockdown, Student-teacher interaction, Digital tools

## Abstract

In this paper we examine the impacts of the global pandemic in 2020 on different levels of education system, particularly looking at the changes in teaching practice. The health emergency caused closure of schools, and online distance education became a temporary solution, creating discomfort for many teachers for whom this was the first time engaged with online education. In our research we investigated two important dimensions, namely, how technology was used and what the newfound distance meant in terms of the teacher-student relationship. The article offers insights into experiences of teaching from lockdown reported by 41 teachers at primary, vocational and higher education level in the region of Vaud, Switzerland. This comparative qualitative research has provided an opportunity for an in-depth analysis of the main similarities and differences at three distinctly different educational levels and a possibility to learn more about common coping practices in teaching. The study gives a contribution to a lack of comparative studies of teacher experiences at different educational levels. Results show two dimensions in handling the lockdown crisis: mastering the digital tools and the importance of student–teacher interaction. Whilst the interviewed teachers largely overcame the challenges of mastering digital tools, optimizing the quality interaction and ensuring the transactional presence online remained a problem. This indicates the importance of the social aspect in education at all levels, and implies that teacher support needs to expand beyond technical pedagogical knowledge of online distance education.

## Introduction

Social interaction and the more affective aspects of distance education have long been central to discussions around the potential efficacy of learning at a distance. Back in 1983, Holmberg posited that, when physically separated, a personal relationship between teacher and learner is still essential for learner motivation and consequently learning. Since then, much research has addressed this issue, but in the majority of cases the focus has been on pupil needs and reactions (Stein et al., 2005). In this paper we look at the teachers’ end of this relationship, and more precisely, question whether teachers need a satisfying social interaction to feel fulfilled in their role as a teacher, as well as how well the increasingly sophisticated technological tools can substitute in-person teaching.

Furthermore, whilst distance education has indeed been used with all ages (Katz, 2002; Passey, 2000) much research around the subject has addressed individual projects across one specific age range (Kotsiantis et al., 2004). The comparative work in this area has focused on comparing distance education with face-to-face education (Sadeghi, 2019), usually having the spotlight on the end results of the process, and from a learners perspective (Mahmood et al., 2012). Yet, not much research has been done to understand how the challenges and opportunities in distance education compare at different levels with different students. Hence, we have set our efforts to examine the common and diverging points in teachers’ experiences at different levels from which we can learn about teaching as a unique profession.

Our study offers insights into how digital technologies support education at different levels, by tapping into understanding teachers’ experiences of teaching with digital tools in forced online distance education caused by COVID-19 lockdown in Spring 2020. The aim was to qualitatively examine the experiences of teachers at primary, vocational education and training (VET) and higher education (HE) level. These three perspectives include extremely different pedagogical approaches, learners’ backgrounds and degrees of developmental maturity. Hence, our intention was to understand the impact of distance teaching in lockdown by reflecting on these two main research questions:

1. What are the similarities and differences in teachers’ experiences of distance education at different educational levels, from the perspective of technology used and from the perspective of teacher-student relationship?
2. What can we learn from comparative perspectives of teaching under COVID-19?

The research was conducted in Switzerland, in the canton Vaud. Due to its exploratory nature, the research was developed using a qualitative interpretative framework, with online semi-structured interviews as the main tool for data collection. By answering the two research questions, this article sheds light on common challenges of teachers using technological tools in education, particularly from the perspective of social interaction between teachers and students. In our results, we present the different tools teachers have used during the lockdown and discuss the commonalities and differences in teaching experiences as they are impacted by student maturity and independence. This leads us to explore the teacher-student relationship as a significant aspect of teaching at all levels of education.

The structure of the paper will provide perspectives on previous literature in the domains of distance education, online education, and a more recent input on forced online distance education. We continue with a section which examines teacher-student relations from the perspectives of available studies at different educational levels. This is followed by a section on context, and one dealing with our methodological approach. Results are provided in two sections, one focused on technological tools and the other on the interaction between teachers and students. This leads to the discussion part, where we examine the similarities and differences and conclude with our take on lessons learnt.

## Literature perspectives

Distance education, defined by Simonson et al. (2011, p. 126) as, “institution-based, formal education where the learning group is separated, and where interactive telecommunications systems are used to connect learners, resources, and instructors” has been around for a long time (Holmberg, 2005). This definition offers two points of departure, first being the use of telecommunications systems and the second referring to the separation of learners, resources and instructors. Furthermore, it is also important to keep in mind that most pedagogical experimentation with distance education is done as a voluntary decision, implemented with teachers and learners who agree to the concepts of being separated and using technological telecommunication tools. In such conditions, studies have shown that there are considerably less differences in student achievement when compared with the traditional, in-person setting. For example, Duvall and Schwartz (2000) noted that there are no significant differences between achievement in online and traditional settings, in adult students. However, they also point out that while there were no differences in technology-adept students and those who are not, ill-designed distance education can have a negative impact on the learning experience which mainly relies on capacities of instructors.

Until recently, we have rarely witnessed large-scale, across the board distance education being practiced in public education for an extended period of time, without a voluntary agreement and preparedness of all parties involved. In their recent study, Dolenc et al. (2021) notice that due to the pandemic, educators and students have been pushed into forced online distance education, and this raised a number of shared problems perceived by both groups, including implementation, motivation, hardware and organizational support. Furthermore, their study also concludes that negative views of students and educators outweigh the positive with technology being among most negative categories, “and educators are more negative than students (Dolenc et al., 2021, p. 21). The physical separation brought by the lockdown changed two main aspects for teachers. First, technology needed to be used in order to continue teaching and this for many teachers was a completely novel and unplanned addition to their daily routine. The second aspect implied that teachers and students were separated from each other and from the learning space, and constrained to their home environments which in most cases were far away from functional learning and teaching settings.

In this paper, we look at these two main aspects of forced online distance education, the first being its technological dimension, and the second the teacher-student interaction aspect.

### Continuous challenges of technology-enhanced pedagogy

The accessibility and rapid spread of personal computers across the developed world in early 1990s was often seen as a chance to revolutionize education, making it more transformative in terms of student learning outcomes. While this helped boost the spread of the idea of online education, the notion of distance education has been already present for a while (Sherry, 1995). The combination of the two has been a rising trend, especially for higher education which only recently has become more open to a wider range of students. However, the transformation promised by technological revolution, did not take place in education in as fast and or as widespread as expected (Brown-Martin & Tavakolian, 2014), and while infrastructure and connectivity coverage were generally improved, use of ICT in pedagogy continued to be a challenge for most. This is mainly because beyond pedagogical content knowledge, teachers needed to understand how to design, facilitate and evaluate meaningful online learning experiences (Dolenc et al., 2021; Rapanta et al., 2020). Literature on massive open online courses (MOOCs) shows a similar conclusion; as an educational innovation that threatened to physically change higher education, MOOCs have only moderately modified the game-play. Gil-Jaurena and Domíngez (2018) note that teachers’ roles have not drastically changed while using MOOCs, and that development of online distance education is above all a way to voluntarily get involved with pedagogical experimentation. Flavin (2017) agrees pointing out that the prevalent way of teaching and learning is still through a lecture and that knowledge is still assessed by final exams. This said, even with a good understanding of the potential and the benefits of digital technologies, it has been difficult for teachers at all levels to develop their online teaching methods beyond simple broadcasting, transferring knowledge and preparing automatized tests (Bourne et al., 2005; Law, 2018; Welch & Napoleon, 2015). Furthermore, it has been noted in literature that teaching with technology demands pedagogical change and innovation and the main critique of existing models was that they take little consideration of the pedagogical components (Law, 2018). On the teacher side, this demands an essential change in their everyday work, and Trigwell (2012) notes that positive feelings about changing pedagogical approaches may motivate teachers to experiment further and use student-focused approaches more frequently. Similarly, when pedagogical experimentation caused negative feelings, teachers usually chose “safer” approaches which are often more teacher-centered (Trigwell, 2012).

In addition, while there is not a lot of literature exposing the comparative aspects of teaching and learning with technology, the existing literature does show that online education better suits the more mature, self-directed learners with well-developed learning strategies (Iqbal et al., 2015; Loya et al., 2015). It is natural to conclude that primary school students are less autonomous than those at the higher levels of education, yet challenges have been reported at the higher education level in terms of student self-directed learning as well. In addition, when examining online learning, studies show that learning community, i.e. students learning together, makes a large difference in learning outcomes (Li et al., 2014). As for teachers, engagement with learners and time requirements are some of the largest challenges when developing online courses (Deng et al., 2019).

### Teacher-student relationship

Literature agrees that in the classroom most of the critical factors for effective teaching are the human factors (Azer, 2005; Harris & Sass, 2009). Although technical factors play a crucial intermediary role in distance education, the human factor is widely considered to be equally central to distance education with technical tools acting as a means to enabling effective teaching and learning to take place (Gillies, 2008). The importance of social interaction and the challenge of preserving relationships in distance learning have been pillars of theorizing on the subject for many years. Holmberg (2005) has long asserted that creating a personal relationship with the learner was crucial to distance education success and Moore and Anderson (2003) introduced the concept of transactional distance, namely the psychological distance between teacher and learner, arguing that this could negatively affect the quality of communication between the two. Shin (2003) took this direction a step further, arguing that, in addition to the question of interaction, the social relationships between the different parties engaged in distance learning are equally crucial, and positing that transactional presence, namely the degree to which learners feel connected to teachers, peers and the institution, is an important predictor of successful distance education.

Interpersonal communication practices like immediacy or self-disclosure have been shown to reduce the psychological distance between teacher and students (Andersen, 1979; Mazer et al., 2007). In the context of online learning, Ghamdi et al. (2016) coined the term “e-immediacy” to describe immediacy (verbal and non-verbal communications) in distance learning contexts. This modality affects non-verbal communication (e.g., eye contact, smiling, physical distance, movement, and graphic information), and verbal communication cues such as use of humor, use of the students names or use of emoticons will emerge to replace them (Song et al., 2016). However, immediacy has still been found “to facilitate student-learning experiences in online education” (Song et al., 2016, p. 437). Teacher self-disclosure is also a way to make the students understand that they are interacting with a human being even if they lack non-verbal communication cues (Song et al., 2016).

## Context

**Fig. 1:**
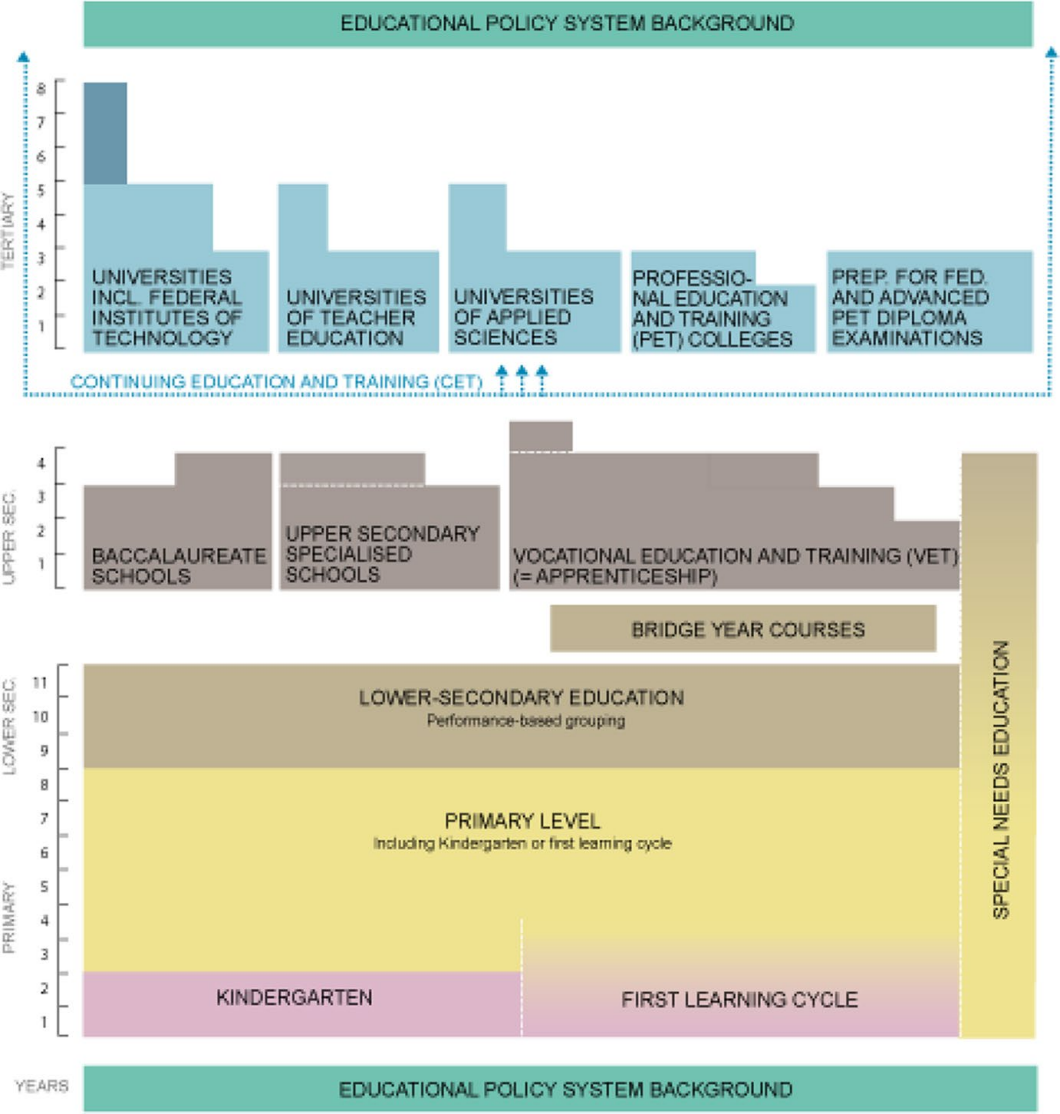
Overview of the cantonal education system

Contextual background plays an important role in all comparative, qualitative studies, and this is not an exception. The current research is placed within three different educational levels in Vaud, Switzerland. Figure 1 provides a visualization of cantonal education system.

For our comparative analysis we have selected three distinct levels: primary level, vocational education and training (VET) and, a university. For the two pre-tertiary levels of education, we targeted institutions at the very beginning of compulsory education and those at the very end, at the upper secondary level, while for the higher education our selection included an institute of technology. With this choice, we aimed at collecting the experiences of teachers whose viewpoints, pedagogical approaches and learning targets in normal times are seen relatively far from each other.

### Lockdown

On the afternoon of Friday March 13 2020, the Swiss Federal Government decided to shut down all schools in the country for an indefinite period of time. This closure affected all levels: from primary to universities and became effective on the following Monday. This unprecedented period lasted 8 weeks in primary schools, and for the remainder of the semester at tertiary level. At the primary level, during the time of school closure, teachers were advised not to introduce any new material into their lecture. Classes resumed with face-to-face teaching in mid-May, with smaller student groups alternating every other day. This modality lasted for 2 weeks. Following this short phase, the school year resumed as before for the last five weeks until the summer holidays. VET schools resumed classes three weeks after primary schools, also with half-classes. Teachers at the VET level were also advised not to include new material in their subjects, but many teachers found that impossible. At primary and VET level, evaluations and exams did not take place at the end of 2019/2020. This was different at HE level where teachers did continue with the planned curricula. The universities continued teaching online for the rest of semester, with some rules becoming looser by the end of the semester and staff being allowed to enter the university building to record teaching segments and laboratory experiments. However, restrictions on student numbers remained in place. Exams did take place for the tertiary level at the end of academic 2019/2020, however due to the circumstances they were postponed and held partly online and partly in-person.

## Methodology

This paper has been developed as a comparative qualitative inquiry, following an interpretivist approach and examining subjective realities through multiple perspectives (Cresswell & Poth, 2018). The methodological approach was devised by the research team comprised of three researchers who later on were also involved in interviewing and data analysis. Since the aim of this paper was to study reactions as experienced from social entities in a specific social setting, a qualitative approach was selected as best fitting, due to the holistic and naturalistic nature of qualitative inquiry (Cresswell & Poth, 2018; Fairbrother, 2007; Merriam & Tisdell, 2016). In addition, qualitative methodology allowed for an open-ended epistemological approach, avoiding preconceived notions (Baker & Edwards, 2012; Cresswell & Poth, 2018; Fairbrother, 2007). This was considered as very important in collecting and analyzing data from highly unprecedented situations, such as the lockdown of the entire education system.

### Comparative framework

**Fig. 2:**
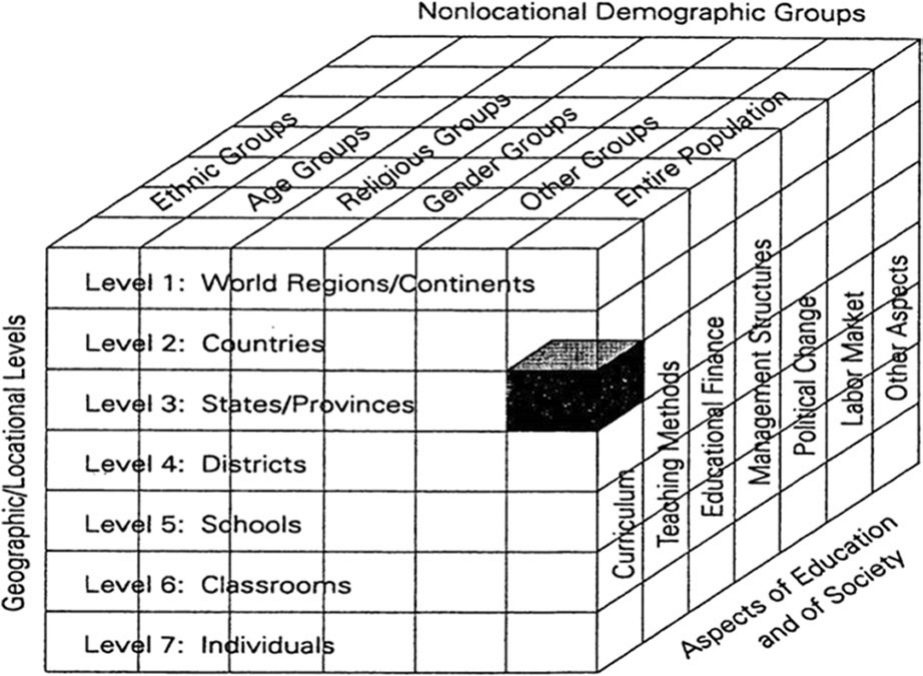
Framework for Comparative Education Analysis. Source: Bray et al., 2007, p. 9

In 1995, Bray and Thomas set the framework for comparative education analyses, and a call was made for more multilevel analyses that would provide multifaceted and holistic studies of educational phenomena (Bray et al., 2007). Their framework, represented in a cube, offers non-extensive perspectives and possibilities of doing such educational research (see Fig. 2).

Following this comparative framework, we set our unit of analysis to be at the specific educational level through qualitatively exploring teachers' experience. The analysis is placed in a particular point of time and specific geographical setting, and the data input is from a single source—teachers.

In considering comparative approaches to education research, Law (2007) notes the importance of not bypassing the pedagogical and human aspects of educational innovation which often focuses too strongly on the use of digital tools. As such, comparisons of pedagogical innovation and change needs to look at ICT tools involved, human factors, primarily teachers and students, and different relations that these categories create including impact on examination, curricular goals and connections with the external world (Law, 2007, p. 328).

With this in mind, our focus in collecting and analyzing data included teacher activities, interactions and collaboration, as well as their reflections on their role. We also took into account types and pedagogical designs of ICT used during the lockdown, and data was also collected on aspects of teachers’ connectedness with students and their interactions with the external world.

### Semi-structured interview guides

Interviews are a widely used tool in qualitative research. The semi-structure nature supports gathering data which might not be easily accessible via most quantitative tools. By using semi-structured interviews, it is possible to provide voice to a certain population, providing an avenue to give viewpoints and tell stories about their experiences. Ultimately, semi-structured interviews provide an opportunity to develop meaning-making of a studied phenomenon (Seidman, 2006).

The semi-structured interview guide used for this study was co-created by the research team who also conducted the data collection. The research team was comprised of three academic researchers, all of whom followed the same pre-agreed interview protocol. The creation of the tool was supported by literature on pedagogical change, including use of tools, challenges, motivation, professional development and emotional elements of change. The interview guide included three main parts, (1) teachers’ reflection on the digital tools and pedagogical matters, (2) reflection on interaction with the students, peers and other stakeholders, and (3) viewpoints on institutional support. The same interview guide was used at all levels of education. Naturally, each interview had an introductory part where teachers could talk about their normal routines and reactions to the lockdown, and a closure where they were asked to share their opinions of the future of education from the perspective of their lockdown experience. We enclose the interview guide as an appendix to the paper.

### Data collection

We conducted our data collection in the period from mid-March to mid-July 2020. Due to health-related restrictions all interviews were done remotely using videoconferencing tools, and the interviews were audio recorded and the recordings were deleted once the data was transcribed. The average length of the interview was approximately 50 min, with shortest being 25 min and longest 80.

In accordance with Cresswell and Poth (2018) we have made interview summaries, memos and reflective notes throughout the data collection period, and these were used in weekly briefings among the research team. The briefing meetings helped in achieving saturation and exploiting of potential topics with interviewees under each of the three segments of the interview. It also supported initial data analysis which was based on memos and researchers’ reflections (Cresswell & Poth, 2018).

It is important to note that only one interview was done through email, while all others were conducted through videoconferencing, mainly using Zoom as a platform. It was of great importance to have the videos on during the interviews, in order to establish a better conversational setting. Remote interviews, such as ones done over the telephone, indicate that interviews tend to be shorter and reduced in theme coverage (Irvine, 2011). Hence, we have paid special attention in mediating the barriers of distance and developing a trusting relationship at the beginning of each interview.

### Participants

The recruitment of the participants was done through teachers voluntarily responding to the call for participation the researchers sent out to the schools. At the primary school and VET levels, the principals were informed as well, however the selection of the teachers was not impacted by the school leadership. At the HE level, a public call was announced to all relevant units working with pedagogical matters, and teachers voluntarily accepted to be interviewed. In terms of the number of interviewees, institutions involved and gender, Table 1 provides an overview.

**Table 1:** Overview of study participants

	Primary	VET	HE
Number of participants	14	17	10
Institutions involved	8	14	1
% of female participants	92%	53%	30%

At the primary school level, the schools were all from the same canton, following the same curriculum. The VET schools involved a whole range of professional education in areas of commers, IT, construction, laboratory work and general studies (languages and history/geography). At the HE level, we interviewed participants of the technological institute which provides education in engineering and architecture. At all levels, the subject taught and years of teaching service were very diverse, ranging from 1–2 years of teaching to beyond 25 years.

Participants for the study were recruited through reaching out to the school leadership who helped spread the word of the study. At all levels, teachers who were contacted consented to voluntarily provide their opinions, viewpoints and describe their experiences for the study.

### Data analysis

The interview data was in all cases transcribed by the researcher team, who also conducted the interviews. Transcripts were safely stored following the ethical protocols and data was pseudonymized to protect interviewees’ identities. In this comparative paper, we selected to create identifiers for our interviewees that will provide information on the level of education followed by a number, as follows P1 for primary school teacher 1, VET1 for vocational education and training teacher 1, and HE1 for higher education teacher 1.

We used NVivo qualitative analysis software and there were two distinct phases in how data was analyzed. In the first round, each researcher has coded their own data inductively in order to look at potential themes and nuances. During this period, the researchers kept informing each other about the ideas arising from the data through frequent meetings. At the end of the first phase, each researcher has developed a summary of their results independently, either in form of a report or a separate article. This allowed for comprehensiveness in how data was analyzed at each level. At the next phase, the researchers exchanged in-dept information on their separate findings and discussed the common points and the joint approach in answering the research questions from a comparative perspective. A common framework was agreed upon, and each researcher had a second look at their own data through deductive coding using a common codebook. This provided the opportunity to move beyond single case analysis to a comparative one.

### Limitations

In order to develop a more complete understanding of our analysis, several limitations in design and data need to be acknowledged. From the perspective of data collection, the current study did not include a wide range of stakeholders’ perspectives, including those of students. Law (2007) points to student practices as one of the key dimensions in achieving holistic analysis, thus this remains a valuable opening for future research.

In addition to this, we take into consideration that we conducted data collection in a specific time period, one that has been severe and stressful for most of our interviewees. While such data is indeed invaluable as it captures and allows for examining a phenomenon in “real-time”, we do acknowledge that should data collection happen at a different time, we would have potentially obtained different viewpoints.

Lastly, due to the conditions in which the research took place, all interviews were conducted by using video conferencing tools. This arguably limited the additional interview data we could harness through noting down the visual cues and non-verbal responses. Nevertheless, almost all interviewees agreed to be interviewed using a video, which helped in establishing a more personal interview interaction.

## Findings

Across three different levels of education, we have categorized our data around the themes that provided main similarities and differences in terms of technological aspects of working under lockdown and from the perspective of their interaction with students. In the following text, we expose our findings following these two categories.

### Technology-enhanced teaching reality

One of the most acute changes brought by lockdown was indeed a surge to use digital tools in education. When comparing our data, we have noticed great differences in how these tools were employed.

In the data from **primary schools**, we see that most teachers used WhatsApp for quick and convenient contact with the parents and with their children through them. Teamup and e-mail were used to share activities or homework. Some teachers also delivered work in the mailbox or, in a few cases, parents were invited to come and retrieve work directly at school.

Interestingly, while primary level teachers have not used teleconferencing tools as we see at other levels of education, WhatsApp and phones were used for recording personalized voice and video messages between teachers and pupils. This asynchronous way of communicating was often adding a personal touch to a difficult emergency situation the teachers, pupils and their guardians found themselves in.

*“For example, I made little videos that I sent to non-native speakers to tell them that if their parents agree, they could send me, from time to time, small videos. Because I tell myself, you can't give them a lot of writing. They need to be able to talk!”* (P5)

At the level of **vocational education and training (VET)** most teachers were at ease with digital tools, and schools used online servers which often were internal. Chat options, such as WhatsApp, were used for quick contact, mainly at beginning, and video-conferencing was the key tool and, according to interviewees, very popular with both teachers and students.

*“It’s great! Yes, it was great because there are so many features: screen sharing, even the whiteboard. You can write on it… if the student has a problem on their side, they can share their screen and I can see where the problem is”* (VET1)

*“The students also thought it was great to have these videoconferences. I could also ask them if they had any questions and if so, they could raise their hands. And then I would pass the floor to them. It allowed for a very full exchange and to remove obstacles that otherwise persist. Most of my pupils, from all my many classes, played the game very well”* (VET6)

Interestingly, the online format had a positive influence on attendance, motivation, participation, and there was a record of some pupils participating more than in the physical class. However, the VET teacher interviewees did stress the importance of having visual contact through keeping the cameras on.

*“Surprisingly, [the participation was] almost better sometimes. I would ask students to put the camera on, I would try to make them put it even if they were in bed, or on their couch”* (VET4)

In terms of asynchronous methods, all interviewees used servers to put documents and retrieve student work, but if internal ones were not accessible, then had to improvise with free online tools. This was quite similar with the HE level interviewees that used an online institutional platform to add video recordings of the lessons, add documents and have asynchronous communication with students. It is worthwhile mentioning that the platform for the HE institution was there before, often used by teachers; hence the distant education mode had only strengthened its consumption.

Another interesting characteristic noticed at the VET level interviews was a number of reported innovations. These referred to during and after distance teaching, by the ways interviewees used online servers, created videos of teaching sequences and capitalizing on questions and exercises during the class time.

*“We ask them to watch the video before coming to class so there’s no demo that we all do together. They’ll have already seen it. They ask questions if they have them. All that to make them a bit more autonomous… And then after the questions we go directly into the exercises, hoping that they’ve done a bit of research, that there’s a bit of curiosity”* (VET3).

Most teachers also noted that they adapted tools to the needs, for instance developing shorter video content to avoid screen fatigue. They also reported using formative tests with individually formed feedback since grading was not allowed. Nevertheless, the tools and approaches were visibly inconsistent which did raise questions among VET teachers for a need for more clarity and direction.

*“The students told me that it was a little bit difficult because each teacher wanted to do things on their own. As a result, for some, there were a lot of differences between the software. They were a bit lost. Some classes said that they wanted everyone to use Claroline to deposit links. Because, each time they need to remember which program is for which teacher. And they had a bit of a trouble with that. Whereas, if everything was centralized on Claroline, they would know that they just need to go there”* (VET3)

Technological tools were less of an issue at the **HE level** and teachers shared only a few troubles, dilemmas and issues with adapting their teaching to emergency distance format. The shock was also quickly mediated centrally by a proactive institutional teacher support services that had rapidly developed and adjusted a number of artefacts, including short guidelines and videos. There were also institutionally organized drop-in pedagogical support sessions and workshops, and individual support offered to anyone who needed it. Additionally, the ease of adapting to digital tools was also reliant on the ability of students to autonomously use technology and, in most cases, possess a personal computer, laptop or tablet.

*“So, what can we say. Obviously, it can be done. This idea of remote learning can be done”* (HE4)

At the HE institution, there was a relatively good level of coherence in what and how IT is used for distance teaching. Evidence from the interviews show that many teachers realized that technology can in a way support their learners, particularly in cases where a video on conceptual or theoretical aspects is recorded and can be accessed and viewed by students at any point as many times as necessary.

*“Now, what I discovered is when I have a video, I can teach with it. I can stop the video and say what is happening in it. Also, I can draw over the video and show the calculations, show the math directly on the object”* (HE6)

Nevertheless, by reflecting on the use of technology, HE teachers did point to the uncomfortable distance in delivering online education. Interviewees noted that most of the synchronous lessons were over Zoom without students having cameras on, which made teachers feel isolated, like speaking to the camera and not to the students. Furthermore, at the HE level, teachers often noticed that the attendance of their live Zoom sessions has dropped and for many this was worrisome, even if students had the access to the recording.

#### Adapting to the new reality

Working with the distance took a great deal of adapting. For many interviewees factors such as getting support in how to use specific technology and how to reach the students was rather important in handling the challenges.

At the **primary level**, interviewees were mostly motivated to take on the challenge of distance learning. They felt the need to do it for their students and for the students' families. However, the interviewed teachers were aware of the fact that something was missing, that they lacked the ability to teach some of the main skills they can teach at school like socializing or living together.

*“And then always the contact. You no longer have that relationship, which is the essence of the job. And then I can no longer manipulate objects with them. Well, I do it by sending pictures. I take pictures of steps to follow, but there is no movement”* (P6)

Hence, for some it was important to support colleagues and students in how best to stay in touch, master the technology, and maintain the need for learning something.

*“We used WhatsApp a lot with colleagues. Each one did according to their skills. There were nice exchanges and offers of help. Some have called each other; some have done Zoom tests. I found that extra nice”* (P9)

*“With my colleague, we supported each other a lot. It happens quite naturally that we find solutions, a system that suits us to transmit to parents. We did it calmly”* (P2)

For several interviewees, the motivation was described in the feeling of responsibility they had towards their class and the challenge to make these moments the most pleasant possible sometime by using technology in a fun, stimulating way.

*“For me, personally, I'm super happy because it poses a new challenge, a new way of teaching and really getting to the bottom of all these digital tools. I have just made a Bookcreator with my students. It's nice!”* (P7)

In the case of **VET schools**, teachers motivated their students to continue learning by providing exercise corrections and formative feedback. In their interviews, they pointed out that it was also important for themselves to maintain a high level of contact with students, and fulfill their teaching role. According to the data, students found self-organization and keeping high motivation difficult, yet, most teachers noted that they think students have gained more autonomy through this experience. There were also external constraints, such as working in social homes or having to look after younger siblings, that impeded students in attending classes and being able to focus on their work.

*“We must not forget that some have private lives that are not necessarily easy. That must be taken into account as well. In the end, we don’t know their family life. There are some who still had to take care of their little brothers. So, I think there is a question of motivation, but also perhaps family duties that others don’t have”* (VET3)

Teachers at the **HE level** have relatively quickly mastered the suggested digital tools used for the emergency distant teaching. Zoom was used both in synchronous and asynchronous mode and know-hows were shared among peers, and to a greater extent by the teaching support units. Nevertheless, interviewees who had smaller children expressed difficulties in working from home and being able to uninterruptedly deliver classes.

*“Combining my professional obligations with having to manage distance learning for my children at home, and having to do with poor or faulty internet connections was really challenging especially at the beginning of the confinement”* (HE10)

*“I have two kids at home and suddenly we’re home all the time, and you have to teach them [the children] and the whole lab”* (HE7)

Furthermore, similarly to the situation at the VET level, HE teachers expressed numerous reflections on their student autonomy, self-directed learning, motivation, lack of socialization and living conditions. Interviewees felt that some students may have suboptimal conditions for learning, and they were concerned that motivation and loss of daily structure might impact the amount to learning. Expressions of worry were very strong especially for the first-year students, a group that generally has a difficult time to adjust and a rather steep learning curve to achieve, particularly with concern that on average 50% of the generation fails the first exam, and has to retake the semester.

*“There is a lot of help for the teachers, but I didn’t see anything for helping the students. It’s maybe natural, they are Internet natives, and maybe they are used to these kinds of tools and maybe we simply need to provide them the lectures and tools and they’ll follow. But I do not think this is the case”* (HE8)

*“Actually, keeping the contact is very important. I think students should not isolate too much. They sometimes live in small studios alone, and their life is in the lab or in the classroom with each other. Suddenly this doesn’t happen anymore and I think this can bring for some a number of problems; anxiety and loss of ability to focus on their studies”* (HE3)

In addition, similar to the primary and VET school teachers, HE teachers were overall worried that they were not reaching the students as they would in physically close environments. This was manifested in teachers explaining that they lack the visual feedback of their students which enables them to understand and "feel" the atmosphere and how much students are attentive and able to follow. The technological barrier made it impossible for teachers to receive the immediate feedback that usually guides their lessons and they were highly bothered by this.

### The student–teacher interaction

The physical displacement of teaching and learning brought a great change to teachers’ routines on all three levels. By talking to teachers, we discovered that, while technology was not always easy to handle, the lack of physical interaction was a much greater concern. Some viewpoints, indicate that lockdown was such a disruption that it questioned the entire idea of what it means to teach.

At the **primary education level,** some of the first impressions brought strong emotions into our conversations, including the most predictive ones like shock, concerns about what to do and worries about the health situation. Yet in a few interviews, teachers talked about a certain excitement that came with the announcement and felt like it was a challenge to take up. Other teachers said they expected it so much that they took the news in a rather trivial way. But even though their feelings were different, most have struggled with the loss of teacher-student relation and invested all their energy to maintain that link, despite the negative feelings.

*“I have the impression that my job was taken from me. What am I going to do? How am I going to do? I couldn't see the end of the tunnel. I felt like I'm going to be there, but I'm going to be useless and all we had to do ... it stressed me a lot!”* (P3)

*“It's true that I am more peaceful since I had contact with the parents and they were able to tell me that for them it was OK, that it was well organized and that they understood, that I see the children doing things at home”* (P11)

In comparison, at the upper levels of education, at **VET and HE**, the first reactions and emotions were much milder. For instance, the main sentiment at the HE level was that a lockdown was expected, even though the interviewees feel they were not appropriately prepared. Reflecting on the change, most of the emotions were related to concerns to student learning, perhaps pointing to an easier absorption of IT tools and stronger focus on difficult, practical, concepts the students need to learn.

*“In the two weeks before the total lockdown, each time I was giving a lecture in the classroom, I was joking with my students. I was telling them that we are here and still can sit in the same room and they can see me moving. And they were always laughing, but then suddenly everything had to close and we all had to start teaching online”* (HE9)

In fact, looking at the data from teachers at **HE level**, we noticed that many were expecting COVID-19 measures to have some form of impact on education, even though there were several that did say it was unexpected. Nevertheless, for all interviewees, regardless of the level of expectancy for moving to online teaching, the transition was not an easy one and teachers admit they were not prepared.

#### Reflecting on the lost connection

The loss of interaction triggered a lot of reflecting on the side of teachers, both from the perspective of how they do their jobs and from the angle of what this interaction means in their work. In some cases, re-establishing this connection was at the core of their attention.

Most teachers at the **primary school level** felt the need to connect with their students during the lockdown. They managed to do that by using technology, through recording personal videos, story-telling, WhatsApp groups, audio messages, e-mail and to some extent video conferencing. However, the interviewed teachers also tried to have more “human” encounters; hence, some interviewees mentioned walking to the homes of their students and waving at the windows, leaving small tokens in the mailbox of their students, and recording pictures and short videos from schools and classrooms, in order to bring the familiar learning environment closer to home-stuck students.

*“So first we said to ourselves: ‘they have to come and get the equipment’. We were there, and it was important to see the parents. I found it nice to know how everyone was doing. And there, we sent an email to the parents to tell them to come at specific day and time”* (P3)

*“We did that every Friday afternoon, telling parents to come between certain hours. We asked them to be respectful of the distance between each other, as well. Frankly, it was a good way to still be able to talk a little bit with the children and, especially, with the parents”* (P1)

*“You see this bond that I was telling you about earlier, which was important to me? Suddenly, it was impossible. That’s why we are putting an envelope into our students’ mailboxes every Monday. They wait for us and give us a little sign at the window. Sometime, they put a note in the mailbox for us!”* (P4)

At the VET level, teachers have relatively easily used teleconferencing to provide individualized support for students who suffered from difficulties in learning. According to the interviewees, these additional “after class” support inputs had a beneficial effect on pupil motivation. Nevertheless, what was noted as heavily lacking even with teleconferencing was the intuitive communication that goes on naturally in a physical class. It was difficult for teachers to see students’ reactions, as the small format of the video call does not provide facial clarity, and quite often the cameras on students’ side were off. Additionally, the teachers experienced a great lack of feedback from students which impacted teachers’ motivation. Furthermore, the physical presence and social energy dynamics of a real class was completely absent in emergency distance education and the teachers described the newfound teaching ambiance lacking when everyone is physically separated behind screens and where there is no physical class space.

*“There is no shared space. As a result, there is relatively little room for intuition and there is no exchange of glances either. There are false glances. Encouragement is also very difficult. Revitalizing is difficult. But it is also possible, like when we returned to face-to-face teaching all students were there, even those who tended to skip classes. We all realized the value of the shared space*” (VET7)

Consequently, teachers noted a greater mental fatigue as big effort was made to scan for reactions and signs.

*“Psychological fatigue in front of the screen is enormous because we try to interpret things the way we are used to when we are in front of a face. But there we can’t because we can’t make eye contact, etc. I said to myself that this has to be taken seriously*” (VET7)

In addition, similarly as at other educational levels, interviewees noticed that some of their students miss the structure of a school day that acts as an external constraint to force them to be present and attentive. But, interestingly, some teachers found the students more efficient as they seemed less distracted by others in class, more willing to work as there was nothing else to do, and for some shy students’ participation in online classes was easier than in the physical class.

*“For some students it was funny because they participated more from a distance than when they were in class, because I think that in class when there are peers, it is more important to talk to friends”* (VET7)

*“I was sometimes rather surprised that they were more efficient being at home, not having the class dynamics that sometimes make them chatter. I was also surprised that they were more efficient being at home*” (VET4)

On the other hand, students were reportedly not at ease with digital tools, and some aspects of the lectures were rather difficult to demonstrate in front of the screen.

*“They are not necessarily comfortable with computer tools because those who are there in their first year, they are a bit new to this world. IT is a bit new too. They do have problems. They had problems with passwords. They didn’t know how to log on or what to do. They were a bit lost!*” (VET1)

A similar reaction was noticed among teachers at the **HE level**. Interaction with students was one of the most common topics of concern. Hence, for the interviewees, the most difficult aspect of distance education was that in most cases teachers would not see the faces of their students, which made them unsure of the effects of their teaching efforts. Teachers would point out that in normal occasions, even if there was no verbal dialogue in the large classrooms, it was at least possible to see the faces and "read" the room, or "feel" the atmosphere. In such a way, they could shift the rhythm, add a joke or a comment, or pose a question that could raise more attention or “wake up” the students.

*“What’s difficult now is the fact that, when you teach you try to get a sense of students and even in a big auditorium you can feel if they follow or not. Sometimes, when I feel they are not following, I do a little joke or a funny historical account to try and get the rhythm back and refocus the students. I tried doing that on the computer and it is very weird”* (HE7)

In the synchronous Zoom sessions, as well as in asynchronous video lectures, teachers could not do much but simply transmit the knowledge and hope it will be helpful for the students. This situation left teachers unhappy about their teaching roles.

#### Social environment and community

While the interaction with students was the most prominent point of topic, teachers did reflect on other human interactions, particularly with their peers and colleagues. Hence, as a last element of exploration, it was interesting to see how the social environment of the newfound lockdown situation influenced teachers in their teaching roles.

The majority of **primary school** interviewees used WhatsApp groups, emails and Zoom to communicate with their peers. The first level of contact was with the colleagues sharing the same classes. They mentioned these relationships as the most important during the first days because the first decisions concerning their classes had to be made rapidly. The second level was the contact with colleagues at the school level. The purpose of this support was mainly pedagogical but also social, as it was important for teachers to maintain the feeling of a virtual teachers' lounge.

*“We have WhatsApp groups between colleagues, sometimes someone starts a discussion like ‘did you understand that?’ And then you realize that other teachers are asking the same questions as yourself. It can be reassuring to say to yourself to know it is not just me asking that question”* (P11)

In addition to this, at the primary school level, institutional support was mostly delivered by mail, although some teachers have noted face-to-face encounters in the first days of lockdown. School leadership was a topic often present in the interviews and most of the teachers reported being told encouraging but vague messages from their principals, such as: “we trust that you are capable”. In fact, the interviewees needed to feel that their superiors were proactive and resourceful, they wanted the support but they also enjoyed the freedom. Interestingly, in several interviews of primary school teachers, spouses was mentioned as a support to the family organization, child care, as well as for emotional support and time management.

At the level of **VET schools**, teachers often received help from colleagues and generally were glad to offer it. Groups were created for sharing ideas and support, both technical and emotional. These were often informal friendship-based discipline groups, but in some cases, there were more formal discipline-based groups. Teacher support to students was however seen as a priority, and students were appreciative of teacher investment. At the institutional level, there were a variety of reactions from interviewees, spanning from having offered concrete support, having given some encouragement, and including not having much support at all.

*“The principal, every week we got an email where she congratulated us for what we were doing. She was really good, surprisingly good*” (VET4)

*“We were very lonely, though, not supported at all*” (VET5)

At the personal level, technical help from younger members of the family was reported as welcome, however, in general mixing of private and professional spheres was experienced as complicated. Video conferences gave a window into personal lives, and homes, which was perceived as an invasion of privacy. At this level of education, teachers also noticed vast differences in digital equipment their students would have available, and the impacts of socio-economic backgrounds and conditions at students’ homes.

*“Having to work often in the kitchen, with their mum hanging around, brothers and sisters coming to watch what’s going on. It’s not the same organization as usual*” (VET5)

At the **HE level**, teachers did receive and offer peer support, and meetings at the faculty or at specific group level were rather common.

*“My colleague, she was horrified to have to teach 220 students through videos. So, I showed her how to make small, easy setup, and it worked*” (HE8)

While the interviewees at HE level did not express a lot of concerns for their own conditions, apart from difficulties with child care, as previously noted, all of them talked in much volume about their concerns for the students, and the potential lack of support the students are experiencing. Teachers shared their understanding of students’ lack of motivation and difficulties with adjusting, particularly pointing to the social aspect of learning at campus. With this regard, many teachers shared a point that students not only come on campus for learning, but the entire education is intertwined with a social aspect of meeting peers and friends, having meals and coffees together and engaging in informal subject-related learning through unstructured friendly conversations. Often, this was also acknowledged as the way to successfully socialize into a specific discipline, and create potential future work- and research-related connections.

## Discussion and conclusions

Our aim in this article was to see teachers’ experiences of teaching under lockdown from a comparative perspective. As such, we were curious to understand what the similarities and differences at three significantly different levels of education were, as well as how we can use this knowledge to inform further research and practice. In a nutshell, wide differences were seen in choices of digital technology and to some extent in the support organized in schools for teaching with online tools. At the primary and to some extend at VET level, commercial widespread tools like WhatsApp and Teamup were more convenient to reach to the students, while at the university level, and to some extent VET, software such as Moodle and Zoom were provided by the institutions. Through this we can notice the preparedness of the institutions for using online teaching formats, but we also argue that the difference in the use of the tools was exaggerated by the relative degree of responsiveness and autonomy of learners, who at primary level are more dependent in terms of using digital devices and studying than those at higher education levels. VET students were more capable of independently using devices but autonomy in learning still seemed a challenge, whilst HE students seemed more capable of handling both the digital and learning autonomy. However, even with such large differences, teachers at all three levels struggled with not having the shared physical space with their students and questioned how best they can fulfil their true roles as teachers.

In order to have an overview of our analyses, we summarize results in a form of a table, where we set out the most important components relating to our two dimensions of interest: technology and human interaction.

From our findings and from the summary in Table 2, we see that teachers at all three levels of education shared a common concern to maintain the core function of their teaching at a distance, even though this function varied according to student age. We see a greater focus on maintaining the relationship between teacher and pupil in the two younger age groups where classes are smaller and the teacher–pupil relationship more personalized compared with HE. We also see a greater focus on transmitting course content in the two older age groups, where the pressure to keep progressing along a defined curriculum was greater than in the primary classes. These differences in core teaching goals exerted an influence on tools used.

**Table 2:** Comparative overview of three educational levels

	Primary schools	VET	HE
Technology			
Aims of ICT use	Aim to connect and maintain closeness	Aim to transmit knowledge, deliver subject content and stay connected	Aim to transmit knowledge, deliver subject content
Tools used	Messaging platforms (e.g. WhatsApp) Mail and letter box distribution	Messaging platforms (e.g. WhatsApp) Video conferencing (e.g. Zoom) Online study platforms (e.g. Moodle)	Video conferencing (e.g. Zoom) Online study platforms (e.g. Moodle)
Uses and functions of digital tools	Maintaining social interaction and communicating with children and their carers about learning contents	Establishing normality, communication of learning content Maintaining social interaction and class community	Weekly communication of the learning content Mix between synchronous and asynchronous
Pedagogical ideas and innovations	Ways of transferring educational games and small activities online Personalized video and voice recordings	Using flipped classroom techniques with the class-time focused on interaction around a previously viewed demo video	Development of short and long video content Preparing exercises and lab work remotely
Teacher-student interactions			
Reactions to distance education	High amount of emotions, concern for students losing the touch with school	Concerns for students in terms of not being able to finish their training properly. Concern for student wellbeing	Concern for students and for the amount of curricula needed to be covered
Interaction	Strong feeling of loss of interaction due to a highly tacit curriculum that cannot be taught remotely	Strong feeling of loss of interaction, due to the type of learning students require at VET level	Strong feeling of loss of interaction, due to the complexity of concepts taught and lack of hands-on learning
Important student factors that influence distance teaching	Parents as main collaborators Home space as an important factor in teaching	Home study conditions for students. Professional or domestic obligations. Ability to study autonomously. Fewer peer distractions	Awareness of students being isolated and the potentially negative impacts on their learning

We also see that, although technology contributed significantly to fulfilling these goals, from the pedagogical point of view teachers at all levels found that the quality of classroom relationship was very difficult if not impossible to achieve at a distance, even in the case of HE, where arguably the lecture format is relatively impersonal. This led to a nostalgia for the classroom on the part of teachers and their students. This finding reflects the argument that human factors are central to online distance teaching and learning (Gillies, 2008). It also supports Shin’s (2003) point that it is not merely “interaction” in the broad sense of the term that counts, but a more indefinable quality of relationship in which learner *and* teacher sense the psychological presence of the other, their availability and connectedness.

In this light, these findings are interesting not only because they highlight the need for further pedagogical and technological advances in distance learning in order to come nearer to capturing this elusive quality of psychological presence, but they also throw the spotlight on an aspect of classroom life that we may in normal times take for granted, that is to say the core human relationships between teacher and students and between students and their role in the learning process.

## Interview guide

Opening questions.

- Tell me about yourself, starting from what subject you teach and for how long?
- What is most important for you in your teaching?
- How were you normally structuring your classes before the pandemic?
- What ICT did you use before in your teaching?

Initial stage.

- How did you feel when the measure about school closure came into force?
- What were your initial thoughts and reactions?
- How did you receive and what sort of information?
- What kind of support did you receive and whom from?
- Did you seek help and whom from?

Current stage.

- What would you describe as the main change to how you have been teach under the social distancing measure?
- What would you describe as challenges in this way of teaching (and learning) now and how would you compare them to the problems you have been facing before?
- Do you see any benefits of this way of teaching (also in comparison to previous ways)?
- What are your thoughts about your students? And what do you see as their reactions?
- What do you think is the most important thing your students should learn in this period?

Implications (current and future).

- What does this change of the way you teach mean to you as a teacher?
- Do you think this change will impact your teaching in the future and in what ways?
- What elements of teaching do you think cannot change and require close social proximity and what are those that can be reimagined?

Closing statements.

- With the current experience, what is the future you see for education and how do you see yourself fitting in it?
